# Dynamics of The Γδtcr Repertoires During The Dedifferentiation Process and Pilot Implications for Immunotherapy of Thyroid Cancer

**DOI:** 10.1002/advs.202306364

**Published:** 2024-01-29

**Authors:** Qing Hao, Ruicen Li, Hancong Li, Shu Rui, Liting You, Lingyun Zhang, Yue Zhao, Peiheng Li, Yuanmin Li, Xinagyu Kong, Haining Chen, Xiuhe Zou, Feng Liu, Xiaofei Wang, Juan Zhou, Weihan Zhang, Libing Huang, Yang Shu, JiaYe Liu, Ronghao Sun, Chao Li, Jingqiang Zhu, Yong Jiang, Tao Wei, Kun Qian, Bing Bai, Yiguo Hu, Yong Peng, Lunzhi Dai, Carlos Caulin, Heng Xu, Zhihui Li, Jihwan Park, Han Luo, Binwu Ying

**Affiliations:** ^1^ Department of Laboratory Medicine West China Hospital, Sichuan University Chengdu Sichuan 610041 China; ^2^ State Key Laboratory of Biotherapy and Cancer Center, West China Hospital Sichuan University and Collaborative Innovation Center Chengdu Sichuan 610041 China; ^3^ Health Promotion Center West China Hospital, Sichuan University Chengdu Sichuan 610041 China; ^4^ Division of Thyroid Surgery, West China Hospital Sichuan University Chengdu Sichuan 610041 China; ^5^ Laboratory of Thyroid and Parathyroid Disease Frontiers Science Center for Disease‐Related Molecular Network Chengdu 610041 China; ^6^ Department of General Surgery, West China Hospital Sichuan University Chengdu Sichuan 610041 China; ^7^ School of Biomedical Sciences The Chinese University of Hong Kong Hong Kong SAR 999077 China; ^8^ Key Laboratory of Transplant Engineering and Immunology, Frontiers Science Center for Disease Related Molecular Network, West China Hospital Sichuan University Chengdu 610041 China; ^9^ Colorectal Cancer Center, West China Hospital Sichuan University Chengdu Sichuan 610041 China; ^10^ Gastric Cancer Center, West China Hospital Sichuan University Chengdu Sichuan 610041 China; ^11^ Division of Gastrointestinal Surgery, State Key Laboratory of Biotherapy, West China Hospital Sichuan University Chengdu Sichuan 610041 China; ^12^ Department of Head and Neck Surgery, Sichuan Cancer Hospital, Sichuan Cancer Institute, Sichuan Cancer Prevention and Treatment Center Cancer Hospital of University of Electronic Science and Technology School of Medicine Chengdu 610041 China; ^13^ Division of Pathology, West China Hospital Sichuan University Chengdu Sichuan 610041 China; ^14^ State Key Laboratory of Systems Medicine for Cancer, School of Biomedical Engineering, Institute of Medical Robotics and Med‐X Research Institute Shanghai Jiao Tong University Shanghai 200230 China; ^15^ State Key Laboratory of Primate Biomedical Research, Institute of Primate Translational Medicine, Kunming University of Science and Technology Yunnan Key Laboratory of Primate Biomedical Research Kunming Yunnan 650500 China; ^16^ Department of Otolaryngology – Head & Neck Surgery and University of Arizona Cancer Center University of Arizona Tucson AZ 85721 USA; ^17^ School of Life Sciences Gwangju Institute of Science and Technology (GIST) Gwangju 61005 Republic of Korea; ^18^ Sichuan Clinical Research Center for laboratory medicine Chengdu Sichuan 610041 China

**Keywords:** γδ T‐cells, dedifferentiation, immunotherapy, thyroid cancer, single‐cell

## Abstract

γδ T cells are evolutionarily conserved T lymphocytes that manifest unique antitumor efficacy independent of tumor mutation burden (TMB) and conventional human leukocyte antigen (HLA) recognition. However, the dynamic changes in their T cell receptor (TCR) repertoire during cancer progression and treatment courses remain unclear. Here, a comprehensive characterization of γδTCR repertoires are performed in thyroid cancers with divergent differentiation states through cross‐sectional studies. The findings revealed a significant correlation between the differentiation states and TCR repertoire diversity. Notably, highly expanded clones are prominently enriched in γδ T cell compartment of dedifferentiated patients. Moreover, by longitudinal investigations of the γδ T cell response to various antitumor therapies, it is found that the emergence and expansion of the Vδ2^neg^ subset may be potentially associated with favorable clinical outcomes after post‐radiotherapeutic immunotherapy. These findings are further validated at single‐cell resolution in both advanced thyroid cancer patients and a murine model, underlining the importance of further investigations into the role of γδTCR in cancer immunity and therapeutic strategies.

## Graphical abstract

A cross‐sectional characterization is performed of γδTCR repertoires in thyroid cancers with gradient differentiation states. Additionally, treatment response‐associated repertoires are investigated by longitudinal analyses, revealing the pivotal role of γδ T cells, particularly the Vδ2^neg^ subset, in thyroid cancer immunotherapy. The findings highlighted their potential as a non‐invasive surveillance indicator that distinguishes between benign and well/less differentiated malignant thyroid nodules.

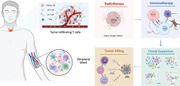

## 1 Introduction

Thyroid cancer is the most common endocrine malignancy, with incidence rates continuing to increase in the past few decades.^[^ ^1^ ^]^ Follicular cell‐derived thyroid cancer mainly includes various histotypes ranging from well differentiated papillary thyroid cancer (PTC) to poorly differentiated (PDTC) and undifferentiated/anaplastic (ATC) thyroid cancer. The patient outcome becomes bleaker as dedifferentiation progresses, with median survival shifting from 10 to 0.5 years. Furthermore, akin to other malignancies, the metastasis of thyroid cancer is often correlated with its progression state.^[^ ^2^ ^]^ Thus, widely invasive PTC (wiPTC) featuring distant metastasis is clearly distinct from typical PTC in terms of differentiation state. Our previous investigations examined the genetic variations, tumor mutation burden (TMB) and heterogeneity of the immune microenvironment during the dedifferentiation process.^[^ ^3^, ^4^ ^]^ Nonetheless, the intricate dynamics of γδ T cell‐mediated antitumor responses during thyroid cancer progression, a pivotal aspect of adaptive immunity, have not been fully elucidated.

γδ T cells are evolutionarily conserved T cells and the main proportion of CD4^−^CD8^−^ double‐negative (DN) lymphocytes. Unlike the well‐characterized αβ T cells that act through human leukocyte antigen (HLA)‐dependent mechanisms, which are associated with TMB,^[^ ^5^ ^]^ γδ T cells are equipped with natural killer (NK) receptors and distinct TCRs composed of γ‐ and δ‐chains. Importantly, they are not restricted by HLA recognition and different subsets straddle innate and adaptive immunity.^[^ ^6^, ^7^ ^]^ Subsets of γδ T cells are defined by TRDV segments usage, with Vδ2 expressing cells typically exhibiting innate‐like properties,^[^ ^8^, ^9^ ^]^ whereas Vδ2^neg^ subsets, such as those expressing Vδ1 and Vδ3, demonstrate adaptive‐like antitumor potential,^[^ ^10^ ^]^ as observed in HLA‐deficient colon cancer.^[^ ^11^ ^]^ Given their role in tumors with low TMB, which are less susceptible to αβ T cell recognition, γδ T cells may offer significant therapeutic promise, particularly in thyroid cancers characterized by a preponderance of DN lymphocyte infiltration.^[^ ^12^ ^]^

To further investigate the antitumor function of γδ T cells, we conducted a cross‐sectional analysis of the γδTCR repertoire across the spectrum of thyroid cancers, ranging from well‐differentiated to undifferentiated types, as well as from localized to widely invasive types. Our study aims to delineate the dynamic landscape of γδTCR repertoires throughout the dedifferentiation process of thyroid cancer, assessing the correlation between γδTCR characteristics and the aggressiveness of malignancies. Additionally, we tracked the γδ T cell response to distinct antitumor therapies in longitudinal cases. The investigation of the TCR repertoire not only enhances our understanding of receptor rearrangements in response to tumor dedifferentiation but also holds great potential to advance cytotherapy for treating lethal thyroid cancers, such as ATC.

## 2 Results

### 2.1 The Distribution of γδTCR Repertoires

**Figure 1:**
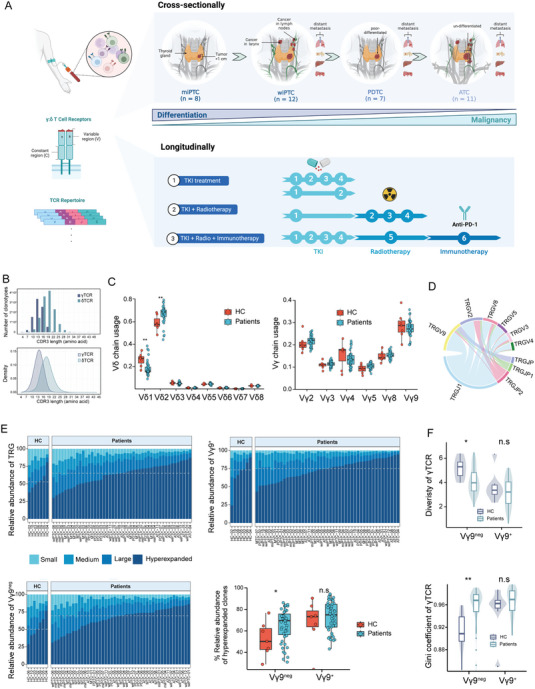
The distribution of γδTCR repertoires. A) Schematic of the experimental design. The top panel represents the cross‐sectional studies from miPTC to ATC. The bottom panel represents the longitudinal studies from three different treatment options. B) The frequency and distribution of CDR3 amino acid lengths. The x axis represents different lengths and the y axis represents the proportion of TCR repertoire (n = 54 samples). C) The frequency usage of TRDV (left) and TRGV (right) gene segments in HC and patients (n = 54 samples). Boxplots indicate the median (thick horizontal line), interquartile range (IQR) spanning 25th to 75th percentiles (box edges), ± 1.5 × IQR (whiskers) and outliers (extreme points). The two‐sided Mann‐Whitney test *p* values are shown. D) V‐J segment recombination abundance in CDR3 junctions for γTCR repertoire (n = 54 samples). E) The occupied homeostatic space of clonotypes in different categories of TRG (top left), Vγ9^+^ (top right) and Vγ9^neg^ (bottom left) (n = 54 samples). Clonotypes are categorized as Hyperexpanded (1–100%), Large (0.1‐1%), Medium (0.01–0.1%), and Small (0–0.01%). The box plot (bottom right) quantified the relative abundance of Hyperexpanded clones in Vγ9^neg/+^ subsets. In boxplots, the median (thick horizontal line), the first and third quartiles (box edges), ± 1.5 × IQR (whiskers) and outliers (extreme points) were showed. The two‐sided Mann‐Whitney test *p* values are shown (n = 54 samples). F) The Shannon diversity (top) and Gini coefficient (bottom) for Vγ9^+/neg^ subsets in HC and thyroid cancer patients (n = 54 samples). In boxplots, the median (thick horizontal line), the first and third quartiles (box edges), ± 1.5 × IQR (whiskers) and outliers (extreme points) were showed. The two‐sided Mann‐Whitney test *p* values are shown. n.s: *p* > 0.05, * *p* < 0.05, ** *p* < 0.01, *** *p* < 0.001, **** *p* < 0.0001.

To delineate the γ‐ (TRG) and δ‐ (TRD) chain repertoires during the dedifferentiation process of follicular cell‐derived thyroid cancer, we collected peripheral blood mononuclear cell (PBMC) samples from 38 treatment‐naïve patients with gradient differentiation states. This cohort included 8 micro PTC cases (miPTCs), 12 wiPTCs, 7 PDTCs and 11 ATCs (**Figure** **1**A). In addition, we obtained PBMC samples from 9 patients with medullary thyroid cancer (MTC), a type derived from parafollicular cells, to cover the entire spectrum of thyroid cancer. In total, 47 PBMC patient samples and 7 healthy controls (HCs) were included in the cross‐sectional analysis.

Moreover, to explore the dynamic alteration of γδTCR repertoires during antitumor treatment, we longitudinally collected PBMCs from 7 patients at 22 time points along the treatment course (Figure 1A). The clinicopathological profiles are summarized in Table S1 (Supporting Information). After quality control, a median of 8793 γTCR clonotypes and 16253 δTCR clonotypes were detected and were highly correlated (*p* < 0.0001; Figure S1A, Supporting Information). The length of the CDR3 sequence followed a Gaussian distribution and presented a bell‐shaped pattern, which was consistent with previous research^[^ ^13^ ^]^ (Figure 1B). The evidence suggested that the γδTCR repertoires identified in our study were reliable for subsequent analyses.

In the pool of 47 patients and 7 HCs, Vγ9 and Vδ2 were the predominant γδTCR sequences in both HC and patient samples (Figure 1C). There were no significant alterations in the use of Vγ segments among patients and HCs. However, patient samples showed an increase in Vδ2 TCR chains and a decrease in Vδ1 chains (Figure 1C). Moreover, we investigated the V‐J gene rearrangement and observed a widely documented CDR3γ sequence, with exclusive utilization of Vγ9 (TRGV9) paired with the JγP (TRGJP) gene fragment (Figure 1D). This Vγ9‐JγP clonotype is the most prevalent semi‐invariant γδT cell population; it features minimal nucleotide trimming and no N‐nucleotide addition, and is shared among adult individuals.^[^ ^9^, ^14^ ^]^ According to our data, the proportion of the Vγ9‐JγP clonotype was not significantly different between HCs and patients (*p* = 0.11; Figure S1B, Supporting Information). Next, we examined the clonal space homeostasis of γδTCR clonotypes. Although the proportion of hyperexpanded clones in the TRG repertoire was comparable between HCs and tumors (52.92% vs 65.16%, *p* = 0.15), a significant difference was observed in the Vγ9^neg^ subset (50.28% vs 69.50%, *p* = 0.045; Figure 1E). Notably, this difference was absent in Vγ9^+^ chains (73.22% vs 74.92%, *p* > 0.05; Figure 1E), which was aligned with their conserved nature.^[^ ^15^ ^]^ Consistently, the diversity and evenness of Vγ9^+^ chains did not differ significantly between HC and tumor samples, but they did in the Vγ9^neg^ population (Figure 1F). Additionally, in the δ‐chain, the proportion of hyperexpanded clones in tumor samples was significantly higher than that in HC samples, independent of Vδ2^neg/+^ (Figure S1C, Supporting Information). Taken together, these results indicate significant discrepancies between HC and tumor samples.

### 2.2 Characterization of γδTCR Repertoires During The Dedifferentiation Process

**Figure 2:**
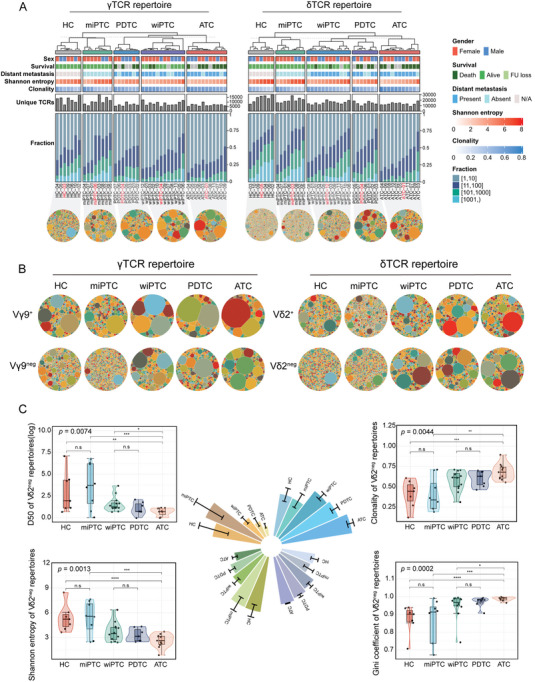
Characterization of γδTCR repertoires during the dedifferentiation process. A) Heatmap showing the landscape of γTCR (left) and δTCR (right) repertoires in different thyroid cancer types (n = 38 samples) and HC (n = 7 samples), with clinically annotated information on the top and immune repertoire features on the bottom. Hierarchical clustering was first applied to each thyroid cancer type using Shannon entropy, generating k dendrograms (child dendrograms). A parent dendrogram was then created based on the mean Shannon entropy of each type. The dashed line discriminates the parent dendrogram from the child dendrograms. Each column represents an individual, with the middle grey bar plots displaying their total number of unique clonotypes. Fraction represents the clonal proportion of the top n clonotypes. The bottom panel shows γδTCR repertoires treemap of representative individuals within each type (highlighted in red). B) Treemaps depicting the Vδ2^neg/+^ and Vγ9^neg/+^ TCR repertoires of various thyroid cancer types, employing representative samples of each type (HC‐10, miPTC‐08, wiPTC‐04, PDTC‐04 and ATC‐01). Each circle represents a unique clonotype and the circle size represents the clonal frequency. CDR3 color is chosen randomly and does not match between plots. C) Graphs depicting the various diversity indicators (DE50, Shannon entropy) and evenness indicators (clonality, Gini coefficient) of Vδ2^neg^ chain in different thyroid cancer types and HC (n = 45 samples). Boxplots indicate the median (thick horizontal line), the first and third quartiles (box edges), and ± 1.5 × IQR (whiskers). The rose plots represent these indicators of Vδ2^neg^ chain (error bar representing the standard error). The *p* values were determined by Kruskal‐Wallis test with Dunn post hoc comparison. n.s: *p* > 0.05, * *p* < 0.05, ** *p* < 0.01, *** *p* < 0.001, **** *p* < 0.0001.

Given the marked differences in γδTCR repertoires between tumors and HCs, we further gained insights into the association between γδTCR and the dedifferentiation of thyroid cancer. We conducted a cross‐sectional comparison among treatment‐naïve samples. Across the progression of dedifferentiation, the incidence of adverse end‐point outcomes (e.g., death, distant metastasis) increased (*p* < 0.0001; **Figure** **2**A). Simultaneously, the unique clonotypes and Shannon diversity significantly decreased, independent of TRG and TRD (both *p* < 0.05) (Figure 2A; Figure S2A, Supporting Information). Visualization of the Vδ2^+/neg^ and Vγ9^+/neg^ repertoires revealed clear oligoclonality in dedifferentiated tumors (Figure 2B). Furthermore, the richness and evenness of each subset were quantified by D50, Shannon entropy, Gini coefficient and clonality (Figure 2C; Figure S2B—D, Supporting Information). The alterations in the Vδ2^+/neg^ and Vγ9^neg^ TCR repertoires were in line with dedifferentiation progression (Figure 2C; Figure 2C,D, Supporting Information). However, no significant alteration was observed in the Vγ9^+^ repertoire (Figure S2B, Supporting Information).

The ATC patients exhibited the highest unevenness and the lowest diversity in Vδ2^+/neg^ and Vγ9^neg^ subsets (Figure 2C; Figure S2C,D, Supporting Information), suggesting that these γδ T cell subsets underwent clonal expansion. This finding was further supported by previous transcriptomic data from differentiated^[^ ^16^ ^]^ and dedifferentiated thyroid cancer patients.^[^ ^17^ ^]^ Regarding δTCR‐associated genes, ATC cases displayed the highest expression of *TRDV2* and *TRDV3* compared to PDTC and PTC patients (Figure S2E, Supporting Information). Furthermore, the diversity, clonality and unique clonotypes of wiPTC and PDTC showed no statistically significant differences, revealing a comparable distribution of clonal proportions between them (Figure 2C; Figure S2B—D, Supporting Information). These similar indexes may provide partial evidence for the dedifferentiation of patients with distant metastasized thyroid cancer.

### 2.3 The Similarity and Clustering of γδTCR Repertoires During The Dedifferentiation Process

**Figure 3:**
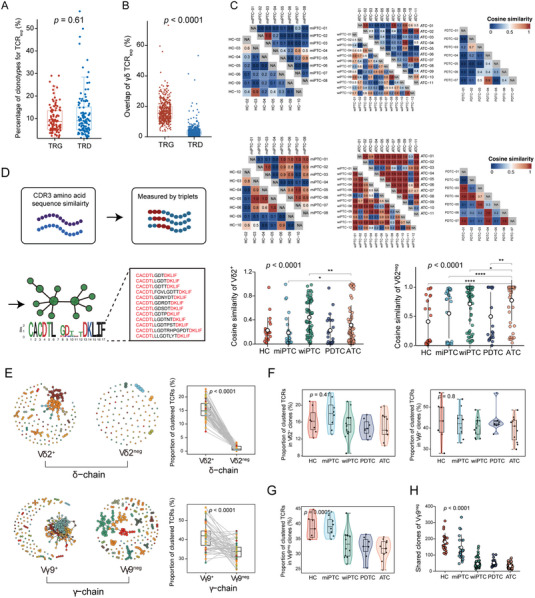
The similarity and clustering of γδTCR repertoires during the dedifferentiation process. A) Boxplot showing that TCR_exp_ occupied 10% unique clonotypes in γδTCR repertoires (n = 54 samples). Boxplots indicate the median (thick horizontal line), the first and third quartiles (box edges), ± 1.5 × IQR (whiskers) and outliers (extreme points). The two‐sided Mann‐Whitney test *p* value is shown. B) Boxplot showing the intra‐type overlap of γδTCR_exp_ (n = 54 samples). The overlap was measured by Immunarch. Boxplots represent the median (thick horizontal line), the first and third quartiles (box edges), ± 1.5 × IQR (whiskers) and outliers (extreme points). The *p* value was determined by two‐sided Mann‐Whitney test. C) Cosine similarity was calculated between each intra‐type individual and the values were indicated by heatmap (Top panel: Vδ2^+^; Middle panel: Vδ2^neg^); bottom panel: comparison of cosine similarity between various cancer types (left: Vδ2^+^ and right Vδ2^eng^) (the Kruskal‐Wallis test with Dunn post hoc comparison *p* values are shown). Each point represents a comparison between two individuals from the same tumor type (n = 191 total comparisons). Line indicates mean ± SD. D) Calculating the pairwise CDR3 similarity and constructing the similarity network. A single cluster is composed of a set of similar CDR3 sequences, in which the amino acid sequences are depicted to the recognition motifs. E) Left panel: the visualization of TCR cluster. A representative sample (ATC‐10) was used to shown the clustering of Vδ2^+^ (top left), Vδ2^neg^ (top right), Vγ9^+^ (bottom left), and Vγ9^neg^ (bottom tight) TCR_exp_. Each circle represents a unique clonotype and each cluster is represented by the same color. Right panel: the proportion of cluster (see Methods) between top 500 Vδ2^+/neg^ (top) and Vγ9^+/neg^ (bottom) TCR_exp_ (n = 54 samples). Boxplot panel shows the median (thick horizontal line), first and third quartiles (box edges) and 1.5 × IQR (whiskers). Each point represents an individual. The *p* value was determined by two‐sided Wilcoxon matched pairs test (paired). F and G) Boxplot of proportion of clustered TCR in different thyroid cancer types in Vδ2^+^ (F, left), Vγ9^+^ (F, right) and Vγ9^neg^ (G) clones (n = 45 samples). Boxplots indicate the median (thick horizontal line), the first and third quartiles (box edges), and ± 1.5 × IQR (whiskers). Each point represents an individual. The Kruskal‐Wallis test *p* values are shown. H) The shared clonotypes of Vγ9^neg^ clones among different thyroid cancer types. Each point represents the shared number between two individuals from the same tumor type (n = 191 total comparisons). Line indicates mean ± SD. The Kruskal‐Wallis test *p* values are shown. n.s: *p* > 0.05, * *p* < 0.05, ** *p* < 0.01, *** *p* < 0.001, **** *p* < 0.0001.

It is well established that tumor‐specific T cells are enriched within the set of expanded T cells in tumors owing to clonal expansion and tissue retention of these cells.^[^ ^18^, ^19^ ^]^ In line with previous research,^[^ ^20^ ^]^ the expanded TCR (TCR_exp_) was defined as a clone with an abundance more than 50% of the mean copy number within each repertoire. The TCR_exp_ constituted ≈10% of the unique TCR sequences in both TRG and TRD, with medians of 624 and 1268, respectively. The two chains exhibited comparability (*p* = 0.61; **Figure** **3**A) and accounted for over 90% of productive reads (Figure S3A, Supporting Information), supporting their expanding phenotype.

Given the intertype differences in diversity (Figure 2A), we next investigated repertoire overlap to estimate the fluctuation in similarity along the differentiation gradient. γTCR_exp_ showed a significantly higher overlap frequency than δTCR_exp_ (14.46% vs 2.26%, *p* < 0.0001; Figure 3B), suggesting that the TRG sequences were more public.^[^ ^8^, ^9^ ^]^ Additionally, we noticed that Vδ2^neg^ showed a significantly lower overlap frequency than Vδ2^+^ did (*p* < 0.0001; Figure S3B, Supporting Information), which implied that the Vδ2^neg^ subset was more private, possibly driven by tumor antigens, and aligned with previous studies.^[^ ^10^ ^]^ In contrast, the significant difference observed above was not detected between Vγ9^+/neg^ chains (Figure S3B, Supporting Information). We then quantified the extent of overlap along dedifferentiation progression using cosine similarity. Intriguingly, no significant differences were found in Vγ9^+/neg^ subsets between distinct thyroid cancer types (*p* = 0.12 and *p* = 0.066; Figure S3C, Supporting Information). This result suggests that TRG remained relatively conserved during the dedifferentiation process, which may be attributed to their primary role in innate immunity. In contrast, the cosine similarity of Vδ2^+/neg^ fluctuated significantly during thyroid cancer dedifferentiation progression (both *p* < 0.001; Figure 3C).

Reportedly, the similarity of TCR sequences suggests shared antigen specificity between the receptors during the antigen driving process.^[^ ^21^, ^22^ ^]^ Similar to αβTCR, γδTCR sequences also “converged” due to a similar recognition motif (Figure 3D). Both TRG_exp_ and TRD_exp_ showed significantly higher convergence than randomly selected TCRs (Figure S3D, Supporting Information), highlighting the characteristics of their antigen‐driven responses. Specifically, the convergence of TCR_exp_ was significantly associated with differentiated states, with the convergence of both chains decreasing along the spectrum of dedifferentiation (both *p* < 0.05; Figure S3E, Supporting Information). However, this phenomenon was absent in randomly selected TCRs in both γ‐ and δ‐chain (both *p* > 0.05; Figure S3F, Supporting Information).

Furthermore, the clustering of Vδ2^+^ and Vγ9^+^ subsets was significantly higher than that of Vδ2^neg^ and Vγ9^neg^, respectively (both *p* < 0.001), when the top 500 clonotypes were selected (Figure 3E). Convergent recombination has been claimed to be the mechanistic basis for the public TCR response.^[^ ^23^ ^]^ Therefore, the difference between the Vδ2^+/neg^ and Vγ9^+/neg^ subsets was consistent with the adaptive‐like Vδ2^neg^/Vγ9^neg^ subset evolving advanced surveillance from the innate‐like Vδ2^+^/Vγ9^+^ subset.^[^ ^10^ ^]^ As expected, there was no significant difference in the clustering of Vδ2^+^ and Vγ9^+^ subsets along the dedifferentiation spectrum (*p* = 0.41 and *p* = 0.8, respectively; Figure 3F). However, the clustering of Vγ9^neg^ decreased significantly (*p* = 0.0005; Figure 3G), and this change was accompanied by a gradual reduction in the number of shared clonotypes (Figure 3H). Given the extremely low clustered TCR in Vδ2^neg^ (the median of 0.8%; Figure S3G, Supporting Information), which also supported the privacy of Vδ2^neg^ clonotypes in tumor development, further clustering analysis in this subset was not applicable.

### 2.4 Alteration of γδTCR Repertoires Over The Treatment Course

**Figure 4:**
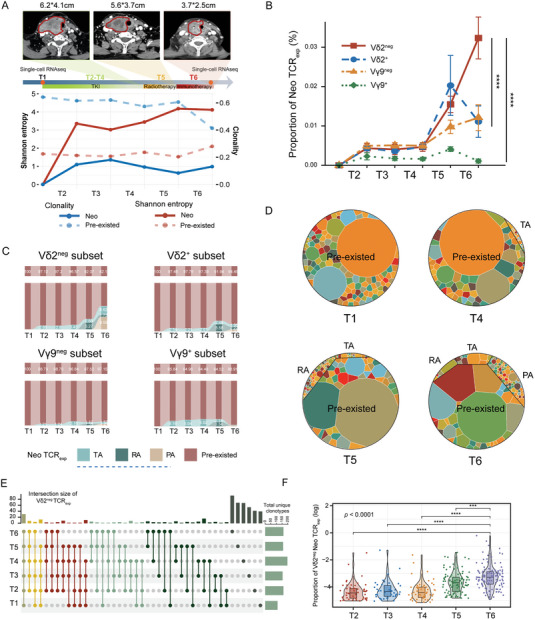
Alteration of γδTCR repertoires over the treatment course. A) Top panel: Schematic representation of the treatment procedure and sampling time for ATC‐06 patient. The green rectangle represents treatment by TKI (corresponding to T2‐T4); the yellow rectangle represents treatment by radiotherapy (corresponding to T5); the red rectangle represents treatment by immunotherapy (corresponding to T6). CT images correspond to the end of each session. Tumors are circled in red. Bottom panel: The Shannon entropy and Clonality of pre‐existed (dashed line) and Neo (solid line) Vδ2^neg^ subsets from patient ATC‐06. B) Line graphs depicting the mean clone frequency of Neo TCR_exp_ ± standard error in Vδ2^neg/+^ and Vγ9^neg/+^ clones at six longitudinal time points from patient ATC‐06. The two‐sided Mann‐Whitney test *p* values are shown. C) Sankey plot indicating the percentage of each type of TCR_exp_ in Vδ2^neg/+^ (top) and Vγ9^neg/+^ (bottom) at six sampling times from patient ATC‐06. TCR_exp_ clonotypes were divided into two categories: Pre‐existed clones and Neo clonotypes (including TA, RA and PA). D) Treemaps of Vδ2^neg^ TCR_exp_ in T1, T4, T5, and T6 from patient ATC‐06. A circle represents a unique clonotype and the circle size represents the clonal size. Each CDR3 color is chosen randomly and does not match between plots. E) Upset plots showing intersections of Vδ2^neg^ TCR_exp_ across six sampling times. If clones were present in multiple sampling times, a line is drawn between those timepoints. The vertical bars are colored by the number of times an intersection is detected. The horizontal bars indicate the number of unique clonotypes per timepoint. F) The boxplot depicted clonal proportion of Neo TCR_exp_ (including TA, RA and PA) in Vδ2^neg^ subsets at each sampling timepoint from ATC‐06. The Kruskal‐Wallis test with Dunn post hoc comparison *p* value is shown (n = 353 total clonal proportion from 225 unique clonotypes). N.s: *p* > 0.05, * *p* < 0.05, ** *p* < 0.01, *** *p* < 0.001, **** *p* < 0.0001.

To decipher the dynamic alterations in γδTCR repertoires in response to antitumor treatments over time, we longitudinally collected PBMC samples from 7 patients who were prescribed tyrosine kinase inhibitors (TKIs) (Mono plan, n = 5), TKI + radiotherapy (Doublet plan, n = 1), and TKI + radio + immunotherapy (Triplet plan, n = 1) (Figure 1A). The treatment course and corresponding sampling points were provided in Table S1 (Supporting Information). Computed tomography presented a clear shrinkage of the ATC tumor size with the Triplet plan (**Figure** **4**A). However, patients with Mono and Doublet plans did not show clear treatment response. To better characterize the dynamic alterations of γδTCR repertoires in response to corresponding treatment course, the TCR_exp_ was divided into pre‐existed and treatment‐responsive Neo TCR_exp_, including TKI‐associated (TA) or(and) radiotherapy‐associated (RA) or(and) PD‐1 treatment‐associated (PA). The specific clone sizes for each patient and course were presented in Table S2 (Supporting Information).

In the Triplet plan (ATC‐06), a longitudinal study of the clone dynamics revealed that Neo TCR_exp_ in the Vγ9^+/neg^ and Vδ2^+/neg^ subsets exhibited similar trends (maintaining lower levels) during the TKI treatment period (T2–T4) (Figure 4B; Figure S4A, Supporting Information). However, following post‐radiotherapeutic PD‐1 treatment (T6), the Neo Vδ2^neg^ subset surged dramatically and was significantly higher than other subsets (Figure 4B). Among them, the increasing portion was primarily composed of PA TCR_exp_ (Figure 4C). Visual presentation of each TCR_exp_ category at these timepoints was shown in Figure 4D. Moreover, the upset plot revealed an increase in distinct clonotypes of Vδ2^neg^ TCR_exp_ at post‐radiotherapeutic PD‐1 treatment (T6) (Figure 4E). Statistically, the clonal proportion at T6 was significantly higher than that at other time points (all *p* < 0.0001; Figure 4F). Therefore, our preliminary findings suggested that post‐radiotherapeutic immunotherapy has an important effect on the expansion of Vδ2^neg^ subset and the establishment of a broader immune repertoire over course.

To confirm our preliminary observation, we longitudinally divided the Triplet plan into 3 phases: TKI phase (T2–T4), TKI+radio phase (T2–T5) and TKI+radio+PD‐1 phase (T2–T6), corresponding to Mono, Doublet and Triplet plan, respectively. The clonal proportion of Vδ2^neg^ Neo TCR_exp_ during TKI phase (T2‐T4) aligned with that of Mono plan (Figure S4B, Supporting Information), and no significant difference in normalized Shannon diversity was detected between them (*p* = 0.1167; Figure S4C, Supporting Information). Similarly, T2–T5 of Triplet plan was also comparable to Doublet plan (p = 0.0571; Figure S4C, Supporting Information). Furthermore, clonal proportion hardly showed significant differences in the entire Mono and Doublet plan (*p* = 0.098 and 0.74, respectively; Figure S4D, Supporting Information), which were notably distinct from that in Triple plan (Figure 4F). And, as expected, normalized Shannon diversity of Vδ2^neg^ TCR_exp_ in Triplet plan was significantly higher than that in Mono and Doublet plan (*p* = 0.026; Figure S4C, Supporting Information). In the view of cross‐sectional comparison, these results supported our longitudinal observations. Given the clinical treatment outcome, it implied that the expansion of Vδ2^neg^ subset may be associated with antitumor effect of Triplet plan.

### 2.5 Single‐Cell Transcriptomics Validation With Human and Murine Samples

**Figure 5:**
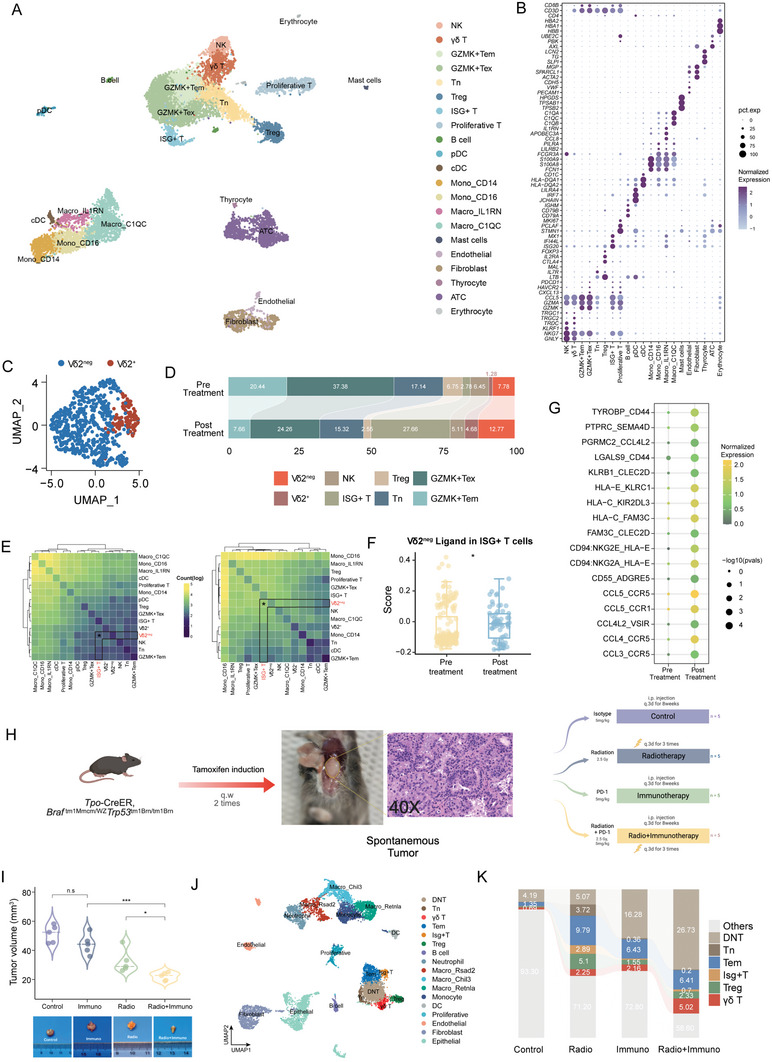
Single‐cell transcriptomics validation with human and murine samples. A) UMAP plot demonstrating the 21 cell types from ATC‐06 patient, colored by cell type. The specified cell types are labeled on the graph. B) Dot plot showing the expression of marker genes of each cell type from ATC‐06 patient. Dot size represents the percentage of cells expression, and the dot color shows the normalized expression level. C) UMAP plot showing the sub‐cluster of γδ T cells. D) The proportion change of T cells between pre‐ and post‐ treatments. E) Predicted cell‐cell interactions on specific cell types using the CellPhoneDB. Left: pre‐; right: post‐treatment. F) Feature scores of ISG^+^ T cell cluster for Vδ2^neg^ T cells ligand in pre‐ and post‐ treatment. The two‐sided Mann‐Whitney test *p* values are shown. G) Pre‐ and post‐comparison of ligand‐receptor cell‐cell communication analysis between Vδ2^neg^ T cells and ISG^+^ T cells. Dot size represents the ‐log10(*p* value), and the dot color shows the normalized expression level. H) Schematic of the experimental design for mice. I) Tumor volumes before and after treatment were compared between different therapies (the two‐sided t test *p* values are shown; n = 5 per treatment group) (top). Representative tumor images of mice in different treatments (bottom). J) UMAP plot showing the 17 cell types from mouse samples, colored by cell type. The specified cell types are labeled on the graph. K) The proportion change of T cells among different treatments. n.s : *p* > 0.05, * *p* < 0.05, ** *p* < 0.01, *** *p* < 0.001, **** *p* < 0.0001.

To investigate the cellular alterations before and after the Triplet plan, we dissected tumor tissues obtained before and after treatment (T1 and T6) (Figure 4A) for single‐cell RNA sequencing (scRNA‐seq). After quality‐control filtering, a total of 13564 cells were obtained and clearly clustered (**Figure** **5**A). Based on the expression of canonical genes, we identified NK cell, 7 T cell clusters (including γδ T cells, GZMK^+^ effector memory T cells (Tem), GZMK^+^ exhausted effector memory T cells (Tex), naïve T cells (Tn), proliferative T cells, regulatory T cells (Treg), and interferon‐stimulated genes (ISG)^+^ T cells); 5 myeloid cell clusters (including monocyte, macrophage, plasma dendritic cells (DC), conventional DCs and mast cells); thyrocyte, thyroid cancer cells, fibroblasts, and endothelial cells (Figure 5B; Figure S5A, Supporting Information). According to previous reports,^[^ ^24^ ^]^ γδ T cells were classified into innate‐like Vδ2^+^ (*KLRC1* ^+^ *GZMK* ^+^ *FCER1G* ^+^) and adaptive Vδ2^neg^ (*TRGC2* ^+^ *IL32* ^+^ *FGFBP2* ^+^) T cell subsets (Figure 5C; Figure S5B, Supporting Information).

Considering the crucial role of T cells in immunotherapy, we further investigated the alterations in the proportions of each T cell subset. The proportions of GZMK^+^Tem, GZMK^+^Tex and Treg decreased; however, the proportion of ISG^+^ T cells and Vδ2^neg^ T cells increased after treatment (Figure 5D). It is well established that γδ T cells exert their antitumor functions through interferon‐γ production.^[^ ^25^ ^]^ A substantial increase in ISG^+^ T cells from 2.78% to 27.66% following treatment (Figure 5D), indicating an activation state possibly driven by TCR‐triggered interferon‐γ or interferons.^[^ ^26^ ^]^ To validate these findings, we utilized CellPhoneDB to infer ligand‐receptor interactions before and after treatment separately. The analysis revealed an apparent increase in communication between ISG^+^ T cells and Vδ2^neg^ T cells after treatment (Figure 5E). Furthermore, the ligand score of Vδ2^neg^ T cells (Table S3, Supporting Information) was significantly upregulated in ISG^+^ T cells after treatment (*p* < 0.05; Figure 5F). The upregulated ligand‐receptor pairs were mainly involved in chemokine (CCL‐CCR) and NK (HLA‐NKG) interactions, such as HLA‐C_ KIR2DL3 and CCL5_CCR5 (Figure 5G). These findings validated the potential role of Vδ2^neg^ T cells in antitumor immunotherapies to some extent, consistent with previous research in various cancers.^[^ ^11^, ^27^, ^28^, ^29^ ^]^

Next, we conducted an animal experiment using genetically engineered (*Tpo*‐Cre, *Braft^m1Mmcm/wz^ TrP53^1Brn/1Brn^*) mouse models to further investigate the potential antitumor role of γδ T cells in post‐radiotherapeutic immunotherapy. The mice, which spontaneously developed ATC after tamoxifen induction, were treated with IgG isotype, radiotherapy, immunotherapy and radio + immunotherapy (combination therapy) (Figure 5H). Consistent with the clinical treatment outcomes observed in ATC patient (Figure 4A), the tumor volumes in the combination treatment group were significantly smaller than those in the other groups (*p* < 0.01; Figure 5I). To further examine the cellular changes in the four treatment groups, we utilized scRNA‐seq and identified 6 T cell subsets, including Isg^+^ T, Tem, Treg, Tn, DN T and γδ T cells (Figure 5J; Figure S5C and D, Supporting Information). Notably, γδ T cells showed a substantial increase in the combination treatment group compared to the control and single‐agent treatment groups (0.69%, 2.25%, 2.16%, and 5.02%, respectively; Figure 5K).

**Figure 6:**
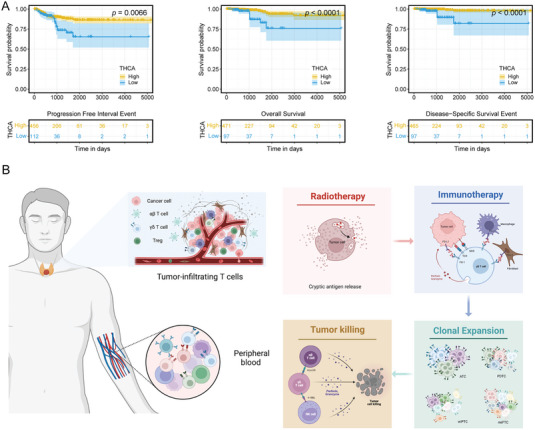
Anti‐tumoral effect of γδ T cells following treatment. A) Kaplan‐Meier survival curves and risk tables for progression free interval (PFI), overall survival (OS), and disease‐specific survival (DSS) in TCGA THCA cohort. The yellow lines represent high group; the blue lines represent low group. Numbers of patients at risk at each time point were shown in the risk table. B) Sketch map showing the process of clonal activation and expansion of γδ T cells.

To further explore the significance of γδ T cells in various cancers, we examined TCGA cohorts and identified a significant association between high expression of Vδ2^neg^ T cell ligands and favorable outcomes in thyroid cancer (THCA) (*p* = 0.0066, < 0.0001 and < 0.0001, respectively; **Figure** **6**A). Similar findings were observed in several other solid cancers (Figure S6, Supporting Information), further supporting the potential antitumoral role of γδ T cells at the pancancer level.

## 3 Discussion

γδ T cells act as compelling effectors of immunotherapy across various cancer contexts, attributed to their antitumor activity independent of tumor mutation burden (TMB) and major histocompatibility complex (MHC) restriction, and possessing dual peculiarity of T cells and natural killer cells.^[^ ^30^ ^]^ It is particularly critical for thyroid cancer, given its distinctive features of a relatively low TMB and a substantial infiltration of DN T cells. However, there is a gap in research concerning the dynamics of γδTCR repertoire in peripheral blood of thyroid cancer patients among divergent differentiated states, following distinct therapy and their relationship to therapeutic efficacy.

In this study, we characterized the γδTCR repertoires in PBMCs from treatment‐naive patients with distinct differentiated states. Throughout the dedifferentiation process, the vast majority of changes were reflected in Vδ2^neg^ subset. The less differentiated state, the more pronounced the expansion of γδ T cell, especially Vδ2^neg^ subset. Early detection and rapid diagnosis of dedifferentiation have been a long‐standing challenge. Our findings suggest that TCR may serve as a potentially sensitive indicator for early detection, and validation in larger cohorts is necessary. Additionally, consistent with evidence that higher tumor‐infiltration of γδ T cells were associated with favorable clinical outcomes, especially Vδ1^+^ cells,^[^ ^27^, ^29^, ^31^, ^32^, ^33^, ^34^ ^]^ we found that Vδ2^neg^ subset may be important for treatment‐responsive T cell. By investigating the dynamics of γδTCR repertoires over the treatment course, we observed that TKI treatment has a limited effect on the increasing of Vδ2^neg^ TCR_exp_, however, post‐radiotherapeutic immunotherapy enhanced the expansion of Vδ2^neg^ TCR_exp_ that may be associated with favorable outcome. The rationale may be interpreted as immune checkpoint inhibitors (ICI) improving γδ T cell proliferation and activation after neoantigens released by radiation.^[^ ^35^, ^36^ ^]^ It suggests that the Vδ2^neg^ TCR repertoires not only mediate antitumor immune responses, but also function as a sensitive surveillance indicator that distinguishes between benign and well/less differentiated malignant thyroid nodules.

Intriguingly, we found that the abundance of Vδ2^neg^ RA TCR_exp_ at T6 substantially decreased compared to T5 (Figure 4C). One possible explanation is that the dominant clones in ICI treatment are the newly emerged distinct clones, rather than the reinvigoration of pre‐existing ones.^[^ ^37^ ^]^ Several reasons may account for this phenomenon (i) exhausted T cells having difficulty reinvigorating antitumor immunity due to their epigenetic stability,^[^ ^38^, ^39^ ^]^ (ii) CXCR5^+^CD8^+^ T cells proliferating only in lymphoid tissues,^[^ ^40^ ^]^ and (iii) the local activation of tumor‐infiltrating lymphocytes (TILs) being insufficient to induce antitumor responses.^[^ ^41^ ^]^

There still are several limitations of this study. First, the generalizability may be limited due to the relatively small sample size in both our cross‐sectional and longitudinal studies. Sampling from the longitudinal cohort is challenging because of poor patient adherence and relative rarity of dedifferentiated thyroid cancer, particularly of PDTC and ATC (1/200000‐1/1000000). Thus, these results observed in longitudinal treatment course should be approached with great caution. Future trials with a larger cohort are required to validate our findings. Thereby, there is an urgent need for a multicenter cooperative effort. Second, we focused on bulk‐level rearrangements of γδ TCR repertoires in our study because of the limitation in tech when study design. Hence, a long‐read single‐cell sequencing which could decipher the TCR information at single cell level is necessary for future exploration.

In summary, early diagnosis of malignancies, especially dedifferentiated nodules, holds paramount importance for thyroid cancer. The sensitive detection ability of Vδ2^neg^ TCR repertoires from PBMC positions them as promising non‐invasive indicator for precision management of thyroid cancer. As the predominant component in DN T cells, γδ T cells, especially the Vδ2^neg^ subset, play a pivotal role in T cell response to thyroid cancer, highlighting their potential as predictive biomarkers for PD‐1 treatment response in advanced thyroid cancer, especially in ATC. Taken together, our study suggests the importance of future studies exploring the role of the γδ T cell repertoire in contributing to the establishment of clinical immunity to dedifferentiated thyroid cancer (Figure 6B).

## 4 Experimental Section

### Clinical Data Collection, Treatment, and Ethics

Clinical and histological information was collected from medical record system. Two experienced pathologists independently reconfirmed sample histology in a blinded manner and classified them according to the eighth edition of the AJCC TNM (American Joint Committee on Cancer Tumor Node Metastasis classification) system. The principles of the Declaration of Helsinki were strictly followed. This study was approved by the Institutional Review Board of West China Hospital of SiChuan University (No. 2021(916)) and registered with ChiCTR2200057655. All patients were recruited at West China Hospital and provided written informed consent. Baseline clinical and demographic characteristics were detailed in Table S1 (Supporting Information). The schematic of this study was illustrated in Figure 1A.

### TCR Sequencing

TCR γ‐chain and δ‐chain sequencing were performed by utilizing whole RNA extracted from PBMC samples. Total RNAs were extracted from PBMC using HiPure Unviersal RNA Mini Kit (Magen R4130‐03). A total amount of 500 ng RNA per sample was used as input material. Sequencing libraries were generated using MultipSeq Custom Panel (iGeneTech, Beijing, China) following manufacturer's recommendations and index codes were added to each sample. The primers were designed based on all rearrangements in the IMGT database, ensuring coverage of all forms in the database. The sequencing library preparation method was a two‐step PCR. The resulting 1st PCR products were purified with magnetic beads and subject to the second round of PCR amplification to add Illumina index and adaptor sequences. The resulting PCR products were purified with magnetic beads and pooled for sequencing with paired end (PE) 2×150 on an Illumina NovaSeq 6000 platform.

### TCR Processing

The fastq files were analyzed using MiXCR.^[^ ^42^ ^]^ MiXCR identified and assembled TCR clonotypes while correcting for PCR and sequencing errors. We filtered these annotated reads with a series of knowledge‐based sieves: (1) removal of any read containing a stop codon in CDR3 region; (2) removal of any read that was out of frame at the CDR3 region. All remaining reads were considered high‐quality and labeled as “‘productive.”’

### Diversity Measure

The Rényi entropy quantifies the diversity of a system:^[^ ^43^, ^44^, ^45^ ^]^

(1) $$\text{Renyi entropy}=\frac{1}{1-\alpha }\log\left(\sum_{i\;=\;1}^{N}p_{i}^{\alpha }\right)$$

where *p_i_* was the frequency of CDR3 sequence i in the TCR repertoire and N was the total number of unique clonotypes; where order α, sets the sensitivity of the diversity index to the abundance of species in the system, ranging from 0 (all species were weighted equally) to infinity (the more weight was given to the more abundant species). When α = 1, the formulation was equivalent to the Shannon entropy:^[^ ^46^ ^]^

(2) $$\text{shannon entropy}=-\sum_{i\;=\;1}^{N}p_{i}\log p_{i}$$

D50 (diversity 50) was calculated in following steps: (1) ranking the clones from largest to smallest by abundance, (2) getting the minimum number of clonotypes were necessary to account for 50% of the total clone size.

### Evenness Measure

Clonality was often used to assess the clonal evenness in TCR repertoire, and was calculated by the following equation (1‐ normalized Shannon entropy):^[^ ^47^ ^]^

(3) $$\text{Clonality}=1-\frac{\sum_{i\;=\;1}^{N}p_{i}\log p_{i}}{\log\left(N\right)}$$

Clonality, which ranges from 0 (all clones with even frequencies) to 1 (a repertoire driven by clonal dominance), with larger clonal expansions resulting in larger clonality values.^[^ ^48^ ^]^

Gini‐coefficient quantifies the evenness of TCR distribution. Gini‐coefficient was calculated by using Gini function in R package DescTools (version 0.99.47) (https://cran.r‐project.org/web/packages/DescTools/index.html).

### Overlap

Overlap frequency was calculated to measure repertoire similarity via the Immunarch package (version 0.9.0) (method = “overlap”).^[^ ^49^ ^]^ It was defined as the size of the intersection divided by the smaller of the size of the two sets.

Cosine similarity analysis was performed to assess the similarity between two TCR repertoires. Based on the overlapping clones in each pair of TCR_exp_ repertoires, two equal‐length vectors were obtained containing the detected number of times in each repertoire. The cosine similarity was then calculated by using

(4) $$\text{cosine similarity}=\frac{\text{TCR1}\cdot \text{TCR2}}{\left|\left|\text{TCR1}\right|\right|*\left|\left|\text{TCR2}\right|\right|}$$

The value ranged from 0 (no overlap) to 1 (complete overlap).

### CDR3 Sequence Clustering

The similarity between each pair of CDR3 amino acid sequences was calculated based on sets of overlapping contiguous amino acid triplets,^[^ ^50^ ^]^ using the stringdot function (with parameters length = 3 and type = “spectrum”) in R package kernlab (version 0.9.31).^[^ ^51^ ^]^ The CDR3 similarity matrix was converted into visualized network plots using the R package iGraph (1.3.1).^[^ ^52^ ^]^ In this network, two CDR3 sequences were connected if the similarity value was >0.8. Therefore, sets of highly related TCR sequences were clustered into one cluster. The clustering network was made up of several clusters.

### Upset Plot

UpSet plots make it easier to visualize intersections of multiple sets than the traditional Venn plots, which were generated by the UpSet function of the ComplexHeatmap package (version 2.8.0).^[^ ^53^ ^]^

### Single‐Cell Sequencing

The Chromium Single Cell 3′ Library & Gel Bead Kit v2 (PN120237), Chromium Single Cell 3′ Chip Kit v2 (PN‐120236), and Chromium i7 Multiplex Kit (PN‐120262) were used according to the manufacturer's instructions in the Chromium Single Cell 3′ Reagents Kits v2 User Guide. The single‐cell suspensions were washed twice with 1× phosphate‐buffered saline (PBS) + 0.04% BSA. Cell number and concentration were confirmed with TC20 Automated Cell Counter. Cells were subjected immediately onto a 10× Genomics Chromium Controller machine for gel beads‐in‐emulsion (GEM) generation. Barcoded complementary DNAs (cDNAs) were prepared using a 10× Genomics Chromium Single Cell 3′ reagent kit (V2 chemistry), which was subsequently recovered, purified, and amplified to generate sufficient quantities for library preparation. Library quality and concentration were assessed using Agilent Bioanalyzer 2100. Libraries were run on the NovaSeq platform of Illumina for PE150 sequencing.

### Single‐Cell Analysis

By the standard pipeline,^[^ ^3^, ^54^ ^]^ Raw bcl files were demultiplexed using mkfastq (Cell Ranger v3.1.0) to generate FASTQ files. FASTQ was mapped to the reference genome (GRCh38 for human; GRCm38 for mouse, respectively) using count application (Cell Ranger v6.0.2) with default settings. Read10 × function from the Seurat package (version 4.1.1) was used to merge all sample data into an aggregate Seurat object. All cells were filtered by several criteria: less than 15% of mitochondrial gene expression, unique gene counts (nFeature_RNA) > 200 and nFeature_RNA < 4000. After filtering, 13564 cells were obtained for human data and 11256 cells for mouse data. Seurat ScaleData and NormalizeData was ran with default parameters. The variable genes were identified by the FindVariableFeatures function of Seurat package. Batch effects were corrected between samples were corrected with RunHarmony function in harmony (version 0.1.0) R package.^[^ ^55^ ^]^ Finally, cells were clustered using the FindNeighbors and FindClusters functions and performed nonlinear dimensional reduction with the RunUMAP (PC = 30, resolution = 1.6 for human; resolution = 0.8 for mouse) function with default settings. Differentially expressed genes (DEGs) between every pair of clusters were identified by using FindMarkers with default settings. For subcluster analysis, the same steps were followed as outlined above (PC = 5, resolution = 0.2).

### Cell‐Cell Communication Analysis

CellPhoneDB was used to analyze the receptor–ligand‐mediated interactions between different cell types.^[^ ^56^ ^]^ This analysis was performed using the CellPhoneDB Python package (version 3.1.0).

### Murine Model Group and Treatment

The animal experiments were approved by the Animal Ethics Committee of West China Hospital (Ethical approval document: 20 230 821 005). As described in the previous report,^[^ ^57^ ^]^ 8‐week‐old TBP (Tpo‐Cre, Braftm1Mmcm/wz TrP53tm1Brn/tm1Brn) mice were induced by i.p. administration of 150 mg k^−1^g tamoxifen dissolved in corn oil for two times. Forty days after induction, mice were randomized into 4 groups: the control group, radiotherapy group, immunotherapy group and combined treatment group (5 mice/group). Control group was intraperitoneally injected with Isotype antibody (5 mg k^−1^g, q.3d for 8 weeks), the radiotherapy group was subjected to sublethal irradiation (2.5 Gy, q.3d for 3 times), the immunotherapy group was intraperitoneally injected with anti‐PD‐1 antibody (BioXCell) (5 mg k^−1^g, q.3d for 8 weeks), and the combined treatment group was subjected to both sublethal irradiation (2.5 Gy, q.3d for 3 times) and anti‐PD‐1 antibody (Bxcell) (5 mg k^−1^g, q.3d for 8 weeks). The mice were sacrificed after 8 weeks of treatment or euthanized due to respiratory distress caused by tumor compression or obvious body weight loss (>20%).

### Survival Analyses and TCGA Cohort

The Cancer Genome Atlas (TCGA) level‐3 RNASeqv2 RSEM gene normalized counts from Illumina HiSeq were downloaded from public TCGA repositories (TCGA Data Portal, https://tcga‐data.nci.nih.gov). These data were then integrated with patients' clinical information. Survival analyses were conducted using survival (version 3.5.3) and survminer (version 0.4.9) R packages in R 4.2.0.

### Statistical Analysis

Data presentation format (e.g., median ± interquartile range (IQR)), number of samples, statistical analysis including multiple comparisons test, and *p* values for comparisons were detailed in the legend for the respective figure panel. The two‐sided Mann‐Whitney test was used for comparisons of two groups; the Kruskal‐Wallis test with Dunn post hoc comparisons test was used for comparisons of three groups or more. Wilcoxon matched‐pairs signed rank test was used for analysis of paired samples. And *p* value < 0.05 was considered statistically significant (n.s: *p* > 0.05, * *p* < 0.05, ** *p* < 0.01, *** *p* < 0.001, **** *p* < 0.0001). Statistical analysis was performed with R (version 4.2.0). Plots were generated using the R package ggplot2. Survival curves were plotted using the R package survminer.

## Conflict of Interest

The authors declare no conflict of interest.

## Supporting information

Supporting Information

Supplementary Table 1

## Data Availability

The data that support the findings of this study are openly available in Genome Sequence Archive in BIG Data Center, Beijing Institute of Genomics, Chinese Academy of Sciences at https://bigd.big.ac.cn/gsa‐human/browse/, reference number 3764.
