# Supplementary material for: Dynamics of The Γδtcr Repertoires During The Dedifferentiation Process and Pilot Implications for Immunotherapy of Thyroid Cancer
Source: Adv Sci (Weinh). 2024 Jan 29;11(13):2306364. doi: 10.1002/advs.202306364 (PMC10987121; doi:10.1002/advs.202306364)
Supplement: Supplementary file 1 — Supporting Information [file ADVS-11-2306364-s002.pdf]

ADVANCED SCIENCE
Open Access

Supporting Information

for *Adv. Sci.*, DOI 10.1002/advs.202306364

Dynamics of The Γδtcr Repertoires During The Dedifferentiation Process and Pilot Implications for Immunotherapy of Thyroid Cancer

Qing Hao, Ruicen Li, Hancong Li, Shu Rui, Liting You, Lingyun Zhang, Yue Zhao, Peiheng Li, Yuanmin Li, Xinagyu Kong, Haining Chen, Xiuhe Zou, Feng Liu, Xiaofei Wang, Juan Zhou, Weihan Zhang, Libing Huang, Yang Shu, JiaYe Liu, Ronghao Sun, Chao Li, Jingqiang Zhu, Yong Jiang, Tao Wei, Kun Qian, Bing Bai, Yiguo Hu, Yong Peng, Lunzhi Dai, Carlos Caulin, Heng Xu, Zhihui Li, Jihwan Park*, Han Luo* and Binwu Ying*

Supplementary Fig. 1 | The distribution of γδTCR repertoires

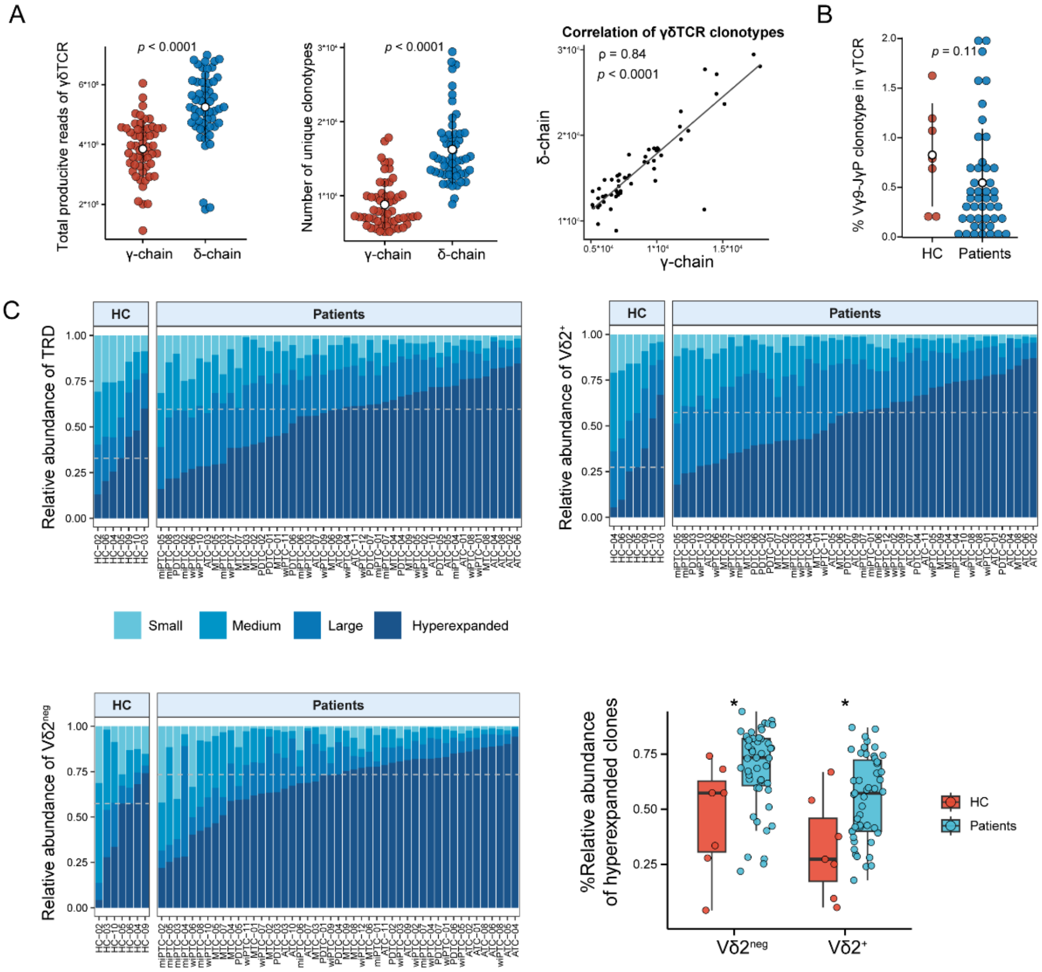

A. Graphs depicting the total productive reads of γδTCR sequences (left), the total number of unique clonotypes (middle) and the correlation between the unique clonotypes of γ-chain and δ-chain (right) in thyroid cancer spectrum (n = 54 samples). Line indicates mean ± SD. Each point represents an individual.The *p* values by two-sided Mann-Whitney test are shown; Spearman rank correlation coefficient and *p* values are shown.

B. The clonal frequency of public Vγ9-JγP clonotypes in HC and cancer patients (n = 54 samples). Line indicates mean ± SD. Each point represents an individual. The two-sided Mann-Whitney test *p* value is shown.

C. The occupied homeostatic space of clonotypes in the different categories of TRD (top left), $V\delta2^{+}$ (top right) and $V\delta2^{neg}$ (bottom left). Clonotypes were categorized as Hyperexpanded (1–100%), Large (0.1-1%), Medium (0.01–0.1%), and Small (0–0.01%). The box plot (bottom right) quantified the relative abundance of Hyperexpanded clones in $V\delta2^{neg/+}$ clones. The two-sided Mann-Whitney test *p* values are shown (n = 54 samples).

n.s: $p > 0.05$, * $p < 0.05$, ** $p < 0.01$, *** $p < 0.001$, **** $p < 0.0001$.

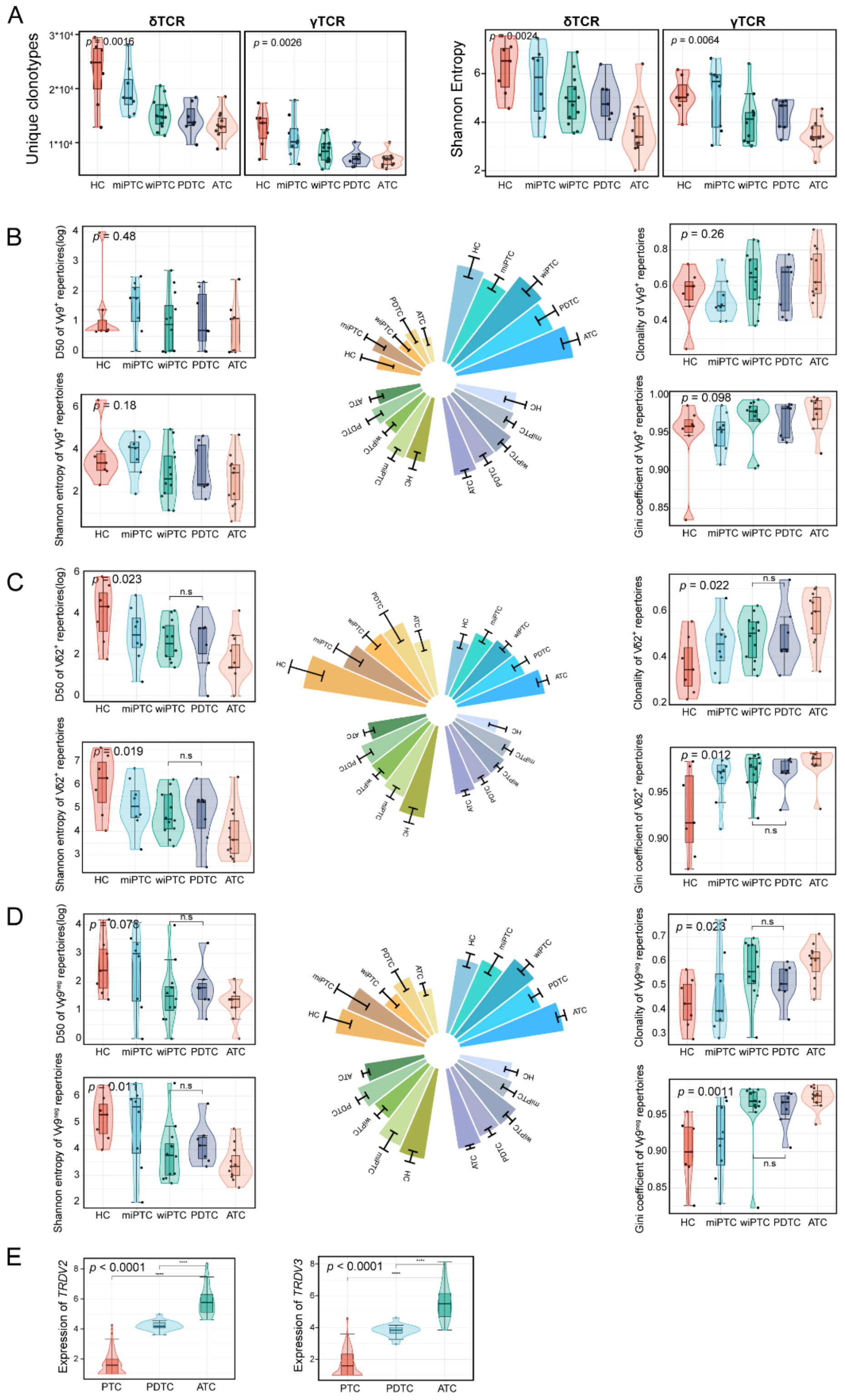

Supplementary Fig. 2 | Characterization of γδTCR repertoires during the dedifferentiation process

A. Graphs depicting the unique clonotypes (left) and the Shannon entropy (right) of γδTCR in different thyroid cancer types and HC. The *p* values were determined by Kruskal-Wallis test.

B, C and D. Graphs depicting the various diversity indicators (DE50, Shannon entropy) and evenness indicators (clonality, Gini coefficient) of Vγ9⁺ (B), Vδ2⁺ (C), and Vγ9^{neg} (D) subsets in different thyroid cancer types and HC (n = 45 samples). Boxplots indicate the median (thick horizontal line), the first and third quartiles (box edges), ± 1.5 × IQR (whiskers) and outliers (extreme points). The rose plots represent these indicators of these chain (error bar representing

the standard error). The Kruskal-Wallis test with Dunn post hoc comparison *p* values are shown.
E. Boxplot of the expression of TRDV2 and TRDV3 in transcriptome sequencing of differentiated (TCGA-THCA) and dedifferentiated thyroid cancers (ATC and PDTC in I. Landa's study). Boxplots indicate the median (thick horizontal line), the first and third quartiles (box edges), ± 1.5 × IQR (whiskers) and outliers (extreme points). The Kruskal-Wallis test with Dunn post hoc comparison *p* values are shown.
n.s : $p > 0.05$, * $p < 0.05$, ** $p < 0.01$, *** $p < 0.001$, **** $p < 0.0001$.

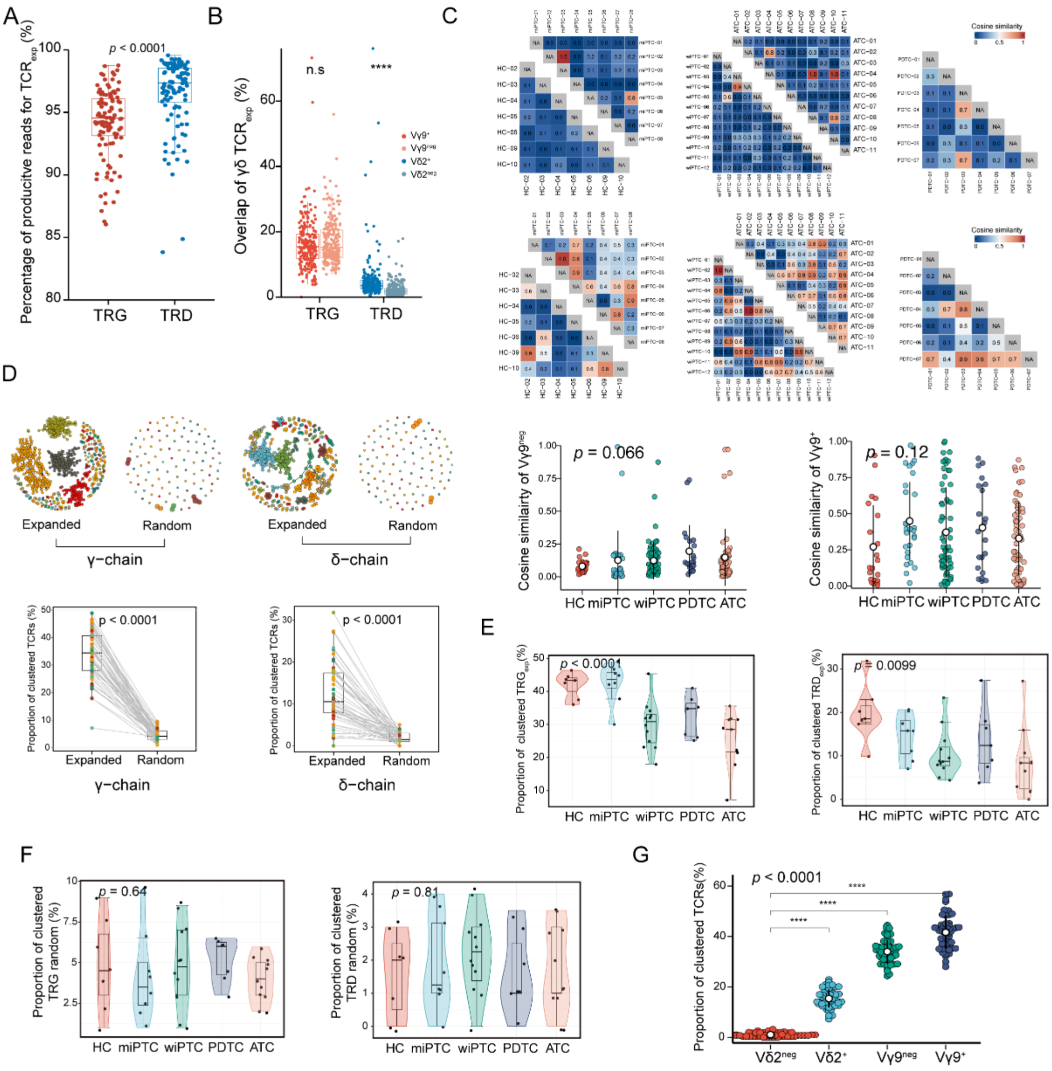

Supplementary Fig. 3 | The similarity and clustering of γδTCR repertoires during the dedifferentiation process
A. Boxplot showing the occupied productive reads of TCR_{exp} in γδTCR repertoires (n = 54 samples). Boxplots indicate the median (thick horizontal line), the first and third quartiles (box edges), ± 1.5 × IQR (whiskers) and outliers (extreme points). The two-sided Mann-Whitney test *p* value is shown.
B. The intra-type overlap frequency of $V\gamma9^{+/neg}$ and $V\delta2^{+/neg}$ subsets. Boxplots indicate the median (thick horizontal line), the first and third quartiles (box edges), ± 1.5 × IQR (whiskers) and outliers (extreme points). Each point represents a comparison between two individuals from the same

tumor type (n = 191 total comparisons). The two-sided Mann-Whitney test *p* values are shown.
C. Cosine similarity was calculated between each intra-type individual and the values were indicated by heatmap (Top panel: $V\gamma9^{+}$; Middle panel: $V\gamma9^{neg)}$; bottom panel: comparison of cosine similarity between various cancer types (left: $V\gamma9^{+}$ and right $V\gamma9^{eng}$) (the Kruskal-Wallis test *p* value is shown). Each point represents a comparison between two individuals from the same tumor type (n = 191 total comparisons).
D. Top panel: the visualization of TCR cluster between TCR_{exp} and randomly selected TCRs by a representative sample (miPTC-03). Each circle represents one clonotype and each cluster is represented by the same color. Bottom panel: Boxplot of proportion of cluster between them (n = 54 samples). Left is γ-chain; right is δ-chain. Boxplot panel shows the median (thick horizontal line), first and third quartiles (box edges), and ± 1.5 × IQR (whiskers). Each point represents an individual. The *p* value determined by two-sided Wilcoxon matched pairs test (paired).
E and F. Boxplot of proportion of clustered TCR in different thyroid cancer types in expanded (E) and random selected sequence (F) in TRG (left) and TRD (right). Boxplot panel shows the median (thick horizontal line), first and third quartiles (box edges), ± 1.5 × IQR (whiskers), and outliers (extreme points). Each point represents an individual. The Kruskal-Wallis test *p* values are shown (n = 45 samples).
G. Dotplot of proportion of clustered TCR in $V\delta2^{+/neg}$ and $V\gamma9^{+/neg}$. Line indicates mean ± SD. Each point represents an individual (n = 54 samples). The Kruskal-Wallis test *p* values are shown.
n.s: $p > 0.05$, * $p < 0.05$, ** $p < 0.01$, *** $p < 0.001$, **** $p < 0.0001$.

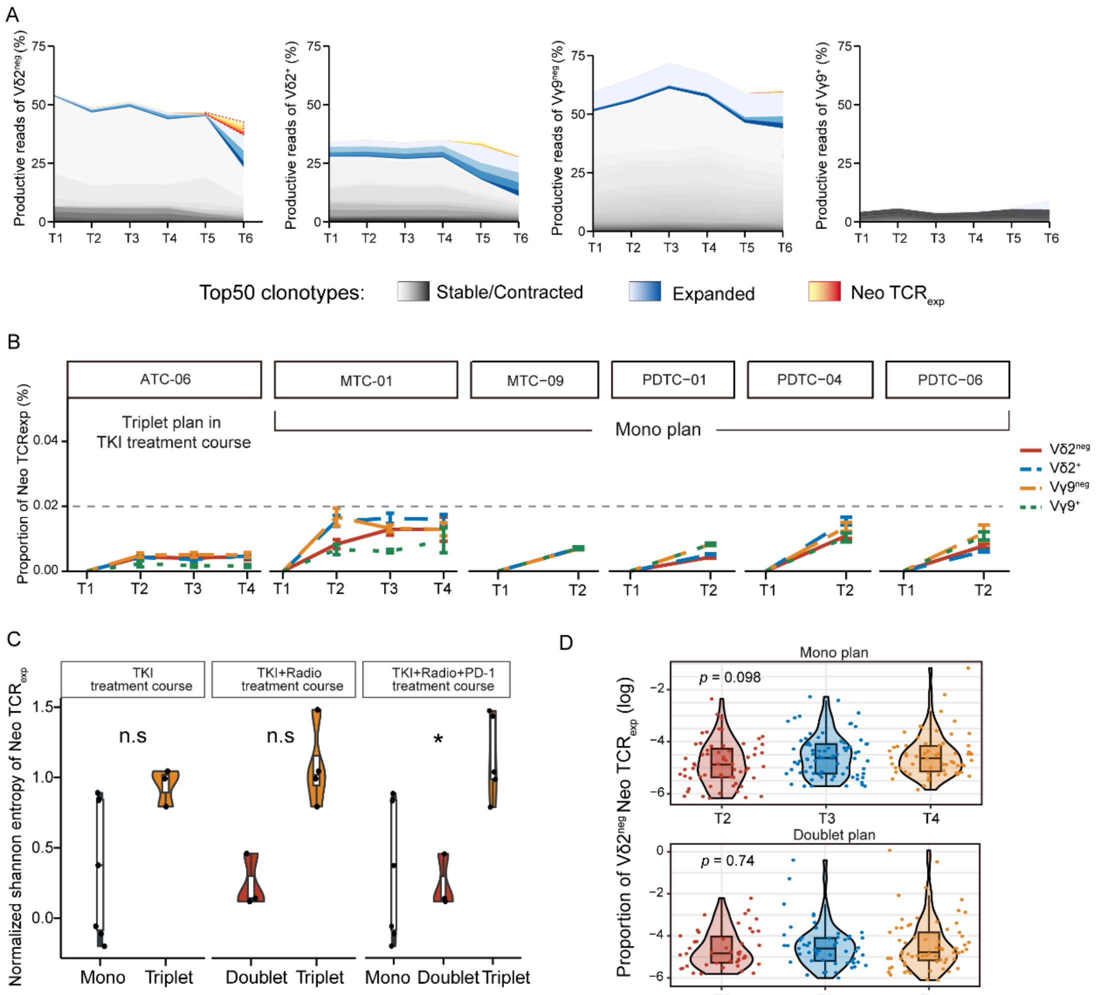

Supplementary Fig. 4 | Alteration of γδ TCR repertoires over treatment course

A. Longitudinal tracking of the 50 most abundant clonotypes in $V\delta2^{+/neg}$ and $V\gamma9^{+/neg}$ T cell repertoires over time.

B. Line graphs depicting the mean clone frequency of Neo TCR_{exp} ± standard error in $V\delta2^{neg/+}$ and $V\gamma9^{neg/+}$ subsets under TKI treatment (Triplet plan only included T2-T4 phase).

C. The cross-sectional comparison of normalized Shannon entropy among multiple patients. In TKI course (left), Mono group included T2 of PDTC-01, PDTC-04, PDTC-06, and MTC-09, and T2-T4 of MTC-01, while Triplet group contains T2-T4 of ATC-06. In TKI+radio course (middle), Doublet group contains T2-T4 of PDTC-02, whereas Triplet group contains T2-T5 of ATC-06. In TKI+radio+PD-1 course (left), Triplet group contains T2-T6 of ATC-06, and other groups remained constant. The normalized Shannon entropy was calculated by $(T_n-T_0)/T_0$. Boxplots indicate the median (thick horizontal line), the first and third quartiles (box edges), ± 1.5 × IQR (whiskers) and outliers (extreme point). The two-sided Mann-Whitney test was used for two group, and the Kruskal-Wallis test for three group.

D. The boxplot depicted clonal proportion of Neo TCR_{exp} in $V\delta2^{neg}$ subsets at each sampling TCR repertoires from Mono plan (top) and Doublet plan (bottom). The Kruskal-Wallis test *p* value is shown.

n.s: $p > 0.05$, * $p < 0.05$, ** $p < 0.01$, *** $p < 0.001$, **** $p < 0.0001$.

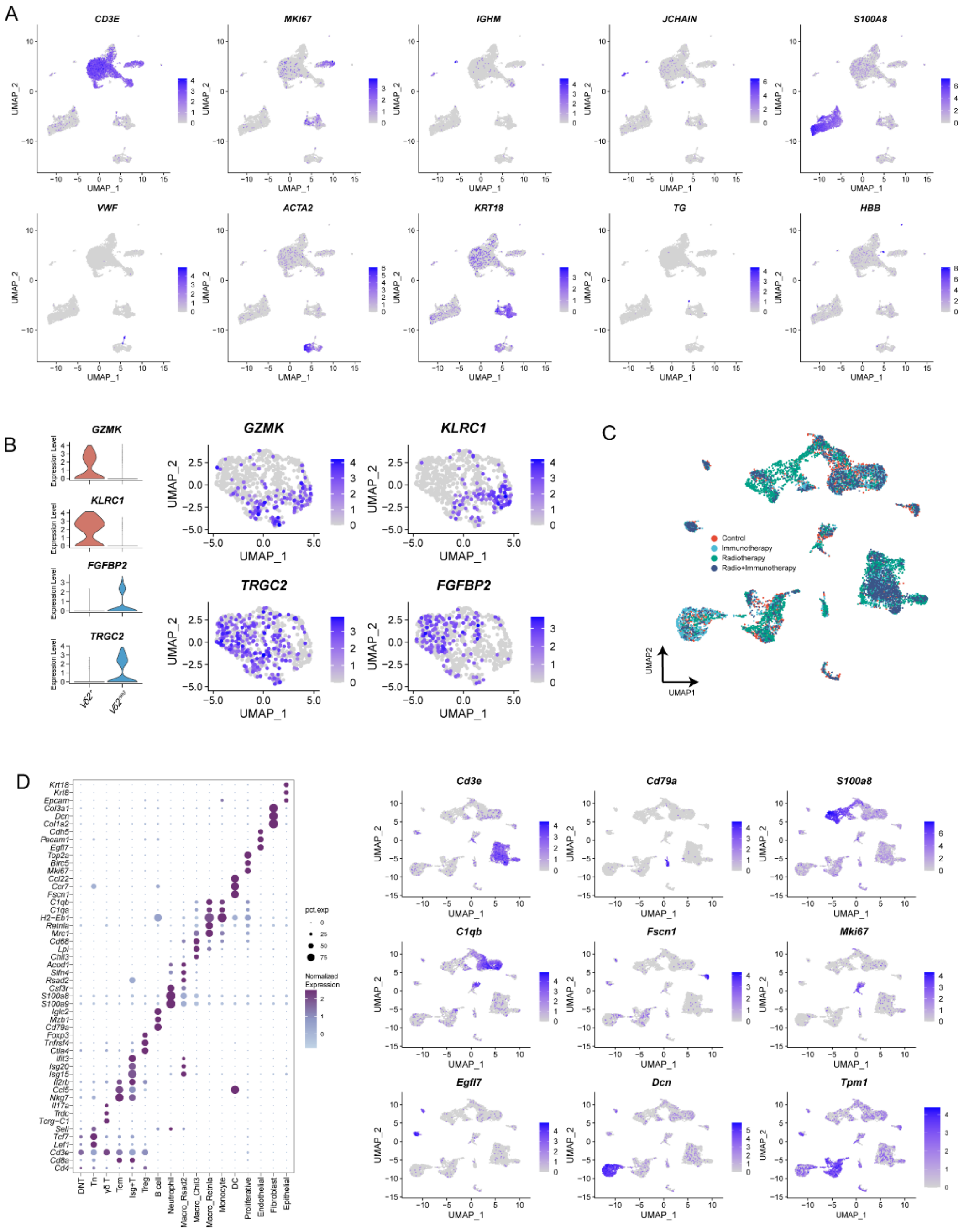

Supplementary Fig. 5 | Single-cell transcriptomics validation with human and murine samples

A. UMAP feature plots showing expression of marker genes by main cell types. Color represents normalized expression of indicated marker genes.

B. Violin (left) and UMAP (right) plots of expression values for specific marker genes in Vδ2^{neg} and Vδ2^{+} T cells. Each dot represents a single cell and colored by normalized expression of indicated marker genes.

C. UMAP plot showing cells from mouse samples. Plot are colored by four treatments.

D. Left panel: Dot plot showing the expression of marker genes of each cell type from mouse samples. Dot size represents the percentage of cells expression, and the dot color represents the normalized expression level. Right panel: UMAP feature plots showing expression of marker genes by main cell clusters. Color represents normalized expression of indicated marker genes.

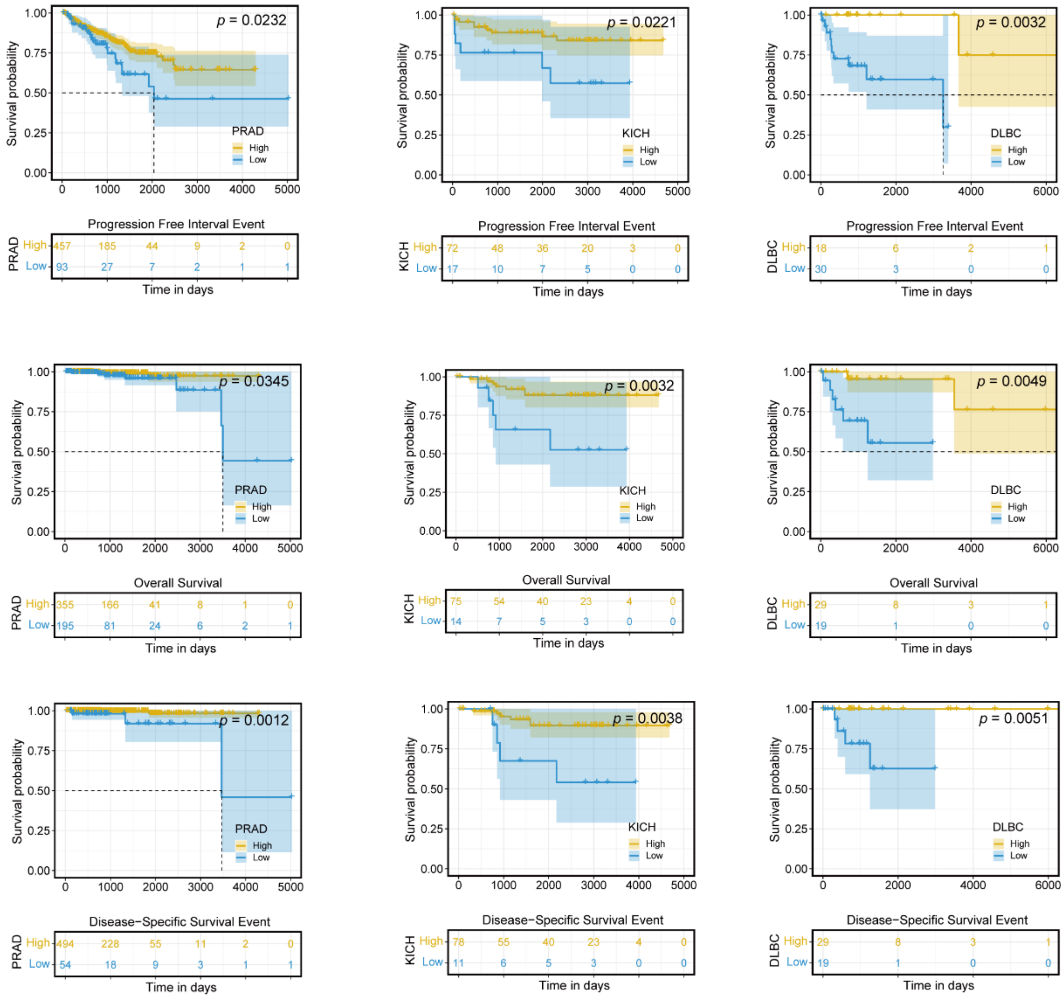

Supplementary Fig. 6 | Survival analysis on TCGA cohort
Kaplan-Meier survival curves and risk tables for OS, DSS and PFI in the TCGA DLBC, KICH and PRAD cohorts with different expression patterns of Vδ2^{neg} T cell ligands. The yellow lines represent high group; the blue line represent low group. Numbers of patients at risk at each time point were shown in the risk table.
